# Restraint of Hæmorrhage during Operations in the Mouth

**Published:** 1874-04-01

**Authors:** E. Andrews

**Affiliations:** Professor of Principles and Practice of Surgery in Chicago Medical College; Chicago, No. 6 Sixteenth street


### RESTRAINT OF HÆMORRHAGE DURING OPERATIONS IN THE MOUTH.

BY E. ANDREWS, M.D., PROFESSOR OF PRINCIPLES AND PRACTICE OF SURGERY IN CHICAGO MEDICAL COLLEGE.

SOME operations in the mouth, particularly staphylorraphy and uranoplasty, are greatly embarrassed and very much prolonged by the following circumstances:

1. The operator and the anæsthetizer cannot both work at the same time, so that they are obliged to alternate — the operator ceasing his work whenever the patient begins to awake, in order that the anæsthesia may be renewed.

2. These operations, considering the small amount of tissue cut, are very bloody, compelling frequent cessation of work, to arrest the hæmorrhage and clear away the blood.

It follows that very often between the suppression of hæmorrhage, the clearing out of blood and mucus, and the repeated re-anæsthetizing, the surgeon gets a vexatiously small proportion of the time in which he can actually use his instruments.

The following successful experiment, to obviate these difficulties, was performed at the suggestion of my friend Dr. Ira Manly, of Markezan, Wis.

The patient being first etherized in the ordinary way, the mouth was held well open, by an instrument which I devised some years ago for this purpose, and have used ever since with excellent results. The paper cone and towel with which the etherization was first accomplished, was now laid aside, and Dr. Manly, taking an ether spray apparatus in his hands (such as is used for local anæsthesia by freezing), directed the spray upon the roof of the mouth; but not with such intensity as to produce freezing. The cold thus produced contracted the vessels, so that I was able to perform uranoplasty with comparatively little delay from hæmorrhage, or accumulation of mucus. At the same time, the patient was constantly inhaling the vapor of the spray, so that the anæsthesia was steadily maintained without interrupting my operative procedures. The result of this method was so admirable, that I desire to call the attention of the profession to its great advantages.

Chicago, No. 6 Sixteenth street.

---

A FORMIDABLE rival to the German Erbswurst has proved its virtues in the Russian expedition to Khiva. It is a biscuit composed one-third of flour of rye, one-third of beef reduced to powder, and one-third of sauer-kraut also reduced to powder. Great relish for the food, and excellent health from its use, have been characteristic of the soldiers throughout the campaign.

